# Antipolymer antibody in Italian fibromyalgic patients

**DOI:** 10.1186/ar2285

**Published:** 2007-09-06

**Authors:** Laura Bazzichi, Camillo Giacomelli, Francesca De Feo, Tiziana Giuliano, Alessandra Rossi, Marica Doveri, Chiara Tani, Russell B Wilson, Stefano Bombardieri

**Affiliations:** 1 Department of Internal Medicine, Division of Rheumatology, University of Pisa, Pisa, Italy; 2 Department of Psychiatry, Neurobiology, Pharmacology and Biotechnology, University of Pisa, Pisa, Italy; 3 Autoimmune Technologies, L.L.C. 1010 Commons Suite 1705, New Orleans, LA 70112, USA

## Abstract

The objectives of the present study were to evaluate the presence of antipolymer antibody (APA) seropositivity in 285 Italian patients affected by primary fibromyalgia (FM) and to verify whether APA levels correlate with disease severity and with cytokine levels.

APA levels were determined on serum samples by an indirect ELISA kit that detects IgG APA. Cytokines (IL-1, IL-6, IL-8, IL-10 and TNFα) were measured by ELISA in plasma. The impact of FM on the quality of life was estimated using the Fibromyalgia Impact Questionnaire, while pain severity was evaluated using a visual analogic scale. Patients were also characterized by the presence of tiredness, stiffness, nonrestorative sleep, anxiety, depression, tension headache, irritable bowel syndrome, temporomandibular dysfunction and Raynaud's phenomena.

Using a cut-off value of 30 U, APA-positive values were detected in 60 FM patients (21.05%) and in 15 healthy control individuals (15.00%) without significant differences among their levels or the percentage of seropositivity. FM patients with moderate and severe symptoms had slightly higher APA levels with respect to patients with mild symptoms. APA-seropositive patients exhibited significant correlations between APA levels and the Fibromyalgia Impact Questionnaire estimate (*P *= 0.042), tiredness (*P *= 0.003) and IL-1 levels (*P *= 0.0072).

In conclusion, APA cannot be considered a marker of disease in Italian FM patients. The presence of APA, however, might permit the identification of a subset of FM patients with more severe symptoms and of patients who may respond differently to different therapeutic strategies.

## Introduction

Fibromyalgia (FM) is a syndrome defined by widespread pain for longer than 3 months and by the presence of ≥11 of 18 tender points [1]. Most FM patients report fatigue, disrupted or nonrestorative sleep, mood disturbances, exercise-induced symptom flares and multiple other syndromes (for example, restless leg syndrome, irritable bowel syndrome and chronic headaches) [1-3]. Physical and emotional health as well as quality of life is often seriously impaired [4-6]. Women are the most affected (9:1 ratio of women to men affected). Like many other clinical syndromes, FM has no single specific feature but represents a complex of symptoms of self-reported or clinical deduction. Unfortunately, there is still no standardized laboratory test to detect FM or to measure its severity.

Researchers have suggested that many of the symptoms reported by women with silicone gel-filled breast implants appear to be similar to those observed in patients with FM [7]. Tenenbaum and colleagues [8] reported that many silicone breast implant recipients produced serum antibodies that recognized what initially appeared to be a high-molecular-weight antigen. After further characterization, it was determined that this antigen was not a protein, but a complex composed of partially polymerized acrylamide. Because of the polymer nature of the antigen, these antibodies have been named antipolymer antibodies (APAs).

Because of the suggested similarities between FM and reported symptoms by patients with silicone gel-filled breast implants, one of us examined the association of APA and FM and found that 47% of patients in a general rheumatological setting in the United States were seropositive for the presence of APA [9]. Consequently, other researchers began to investigate the APA seroactivity in patients with FM, finding controversial data [10,11].

In light of these results, we examined the APA levels in a cohort of Italian FM patients and investigated their association with disease severity and cytokine levels. To determine whether APA results from a generalized autoimmune response, the APA seroreactivity was evaluated also in several autoimmune disease noncase groups including rheumatoid arthritis, Sjögren's syndrome, systemic sclerosis, systemic lupus erythematosus and undifferentiated connective-tissue disease.

## Materials and methods

### Patients

We recruited 285 consecutive patients (270 females, 15 males) affected by primary FM as assessed by the 1990 American College of Rheumatology criteria [12], 40 noncase individuals (16 rheumatoid arthritis cases, two Sjögren's syndrome cases, 16 systemic lupus erythematosus cases, four systemic sclerosis cases, two undifferentiated connective-tissue disease cases) and 100 healthy age-matched and sex-matched subjects. Individuals with a history of silicone gel-filled breast implants or breast surgery were excluded from the study. Written consent was obtained from all participants after a full explanation of the procedure.

For each patient the tenderness at tender points was evaluated by means of the Fischer dolorimeter [13]. A rheumatologist advanced the instrument at a rate of 4 kg/s and the patient was instructed to say when this procedure became painful. The pain threshold was calculated from 18 points, and the tender point count was determined by the number of tender points that had a threshold ≤4 kg/cm^2^. The total fibromyalgic tender point score (right + left) was used in the statistical analysis. Each positive tender point had a pain score between 0 and 3. The Tender Point Index was calculated as the sum of each positive tender point score divided by the total number of tender points. The total pain severity and tiredness were evaluated by a visual analogical scale (0–10).

To estimate the impact of FM on the quality of life, all patients and control individuals received a Fibromyalgia Impact Questionnaire [14,15] consisting of 10 items. The total score ranged from 0 (no impact) to 100 (maximum impact).

All patients were asked whether they had frequently suffered any of the following symptoms [16] in the past 12 months: tiredness, sleep disturbance, anxiety, depression, irritable bowel syndrome, constipation, diarrhoea, Raynaud's phenomenon, paresthesiae, articular stiffness, muscular stiffness, dry eyes, dry mouth, temporomandibular disorders, tension headache, allergy, low back pain, restless leg syndrome, gastroesophageal reflux disease, burning/pain with urination, dizziness, allodynia, traumatic event, blurred vision and sore throat.

We arbitrarily classified the FM severity on the basis of the tender point count: the presence of 11 tender points was considered mild severity, the presence of 14–16 tender points as moderate and the presence of more than 16 tender points was designated severe.

### Antipolymer antibody and interleukin assay

APA levels were determined on serum samples by an indirect ELISA kit that detects IgG APA (Corgenix, Westminster, CO, USA), according to the manufacturer's instructions. Diluted serum samples, calibrator and control samples were incubated in polymer-coated microwells. After the removal of unbound proteins by washing, specific antibodies for human IgG labelled with horseradish peroxidase were added, forming complexes with the polymer-bound antibodies. The bound enzyme–antibody conjugate was assayed by the addition of a single solution containing tetramethylbenzidine and hydrogen peroxide as the chromogenic substrate. Colour develops in the wells at an intensity proportional to the concentration of APA. Results were obtained by reading the optical density at 450 nm. The measure of the optical density was converted in the sample unit using a conversion factor. All the samples were tested in duplicate, and a result of more 30 U was considered positive, as suggested by the manufacturer.

Cytokines (IL-1, IL-6, IL-8, IL-10 and TNFα) were measured by ELISA in plasma (Bender MedSystem, Austria, Vienna). A polyclonal antibody-coating antibody is adsorbed onto microwells, and the cytokine present in the samples or standards binds to antibodies adsorbed to the microwells. A biotin-conjugated monoclonal antibody directed to cytokine is added and binds to cytokine captured by the first antibody. Streptavidin–horseradish peroxidase is added and binds to the biotin-conjugated cytokine. Unbound streptavidin–horseradish peroxidase is removed during a washing step, and substrate solution reactive with horseradish peroxidase is added to the wells. A coloured product is formed in proportion to the amount of cytokine present in the sample. The reaction is terminated by addition of acid and the absorbance is measured at 450 nm. A standard curve is prepared from seven cytokine standard dilutions and the cytokine sample concentration is determined.

### Statistical analysis

Data were analysed by means of nonparametric statistical methods using Kruskal–Wallis analysis of variance, Spearman's correlation, Student's *t *test and the chi-square test. *P *< 0.05 was considered statistically significant.

## Results

**Figure 1:**
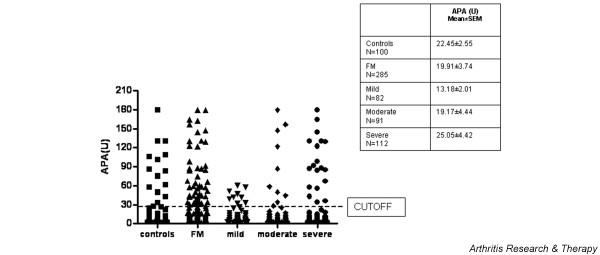
Antipolymer antibody levels in fibromyalgia patients and in control individuals. Antipolymer antibody (APA) levels in fibromyalgia (FM) patients (all patients and patients separated according to disease severity) and in control individuals. SEM, standard error of the mean.

Demographic and clinical data of the 285 FM patients are presented in Table 1. There are no significant differences between the FM subset with positive APA and those FM patients with negative APA. The serum APA level (mean ± standard error of the mean) was 22.45 ± 2.55 U in the FM patients and was 19.93 ± 3.74 U in the control individuals (Figure 1).

**Table 1:** Demographic characteristics, clinical characteristics and antipolymer antibody (APA) levels of 285 fibromyalgia patients.

	All fibromyalgia patients	Patients with APA >30 U	Patients with APA <30 U
Gender (females/males)	270/15	57/3	213/12
Age (years) (median (range))	54 (19–82)	46 (19–74)	56 (20–82)
Tender points	14.29 ± 0.29	13.88 ± 0.83	14.30 ± 0.34
Tender Point Index	2.17 ± 0.04	2.17 ± 0.12	2.19 ± 0.04
Onset disease	9.19 ± 0.60	8.77 ± 1.19	10.11 ± 0.67
Fibromyalgia Impact Questionnaire	58.21 ± 1.22	55.89 ± 2.59	58.46 ± 1.39
Tiredness	6.96 ± 0.17	7.16 ± 0.41	6.88 ± 0.19
Pain	6.80 ± 0.17	6.31 ± 0.42	6.87 ± 0.18

Results expressed as the mean ± standard error of the mean. The Tender Point Index was calculated as the sum of each positive tender point score divided by the total number of tender points.

Considering a cut-off value of 30 U, APA-positive values were detected in 60 FM patients (21.05%) and in 15 healthy control individuals (15.00%) without significant differences in seropositivity among them.

Patients with moderate (19.17 ± 4.44) and severe symptoms (25.05 ± 4.42) had slightly higher APA levels with respect to patients with mild symptoms (13.18 ± 2.01) (Figure 1). No correlations were found between the APA levels of FM patients and the onset of the disease, the tender point counts or scores, or pain.

A negative correlation between APA levels and age was found both in FM patients (*P *< 0.0001) and in control individuals (*P *= 0.0294).

Considering the subset of FM patients with positive APA, significant correlations were found between APA levels versus the Fibromyalgia Impact Questionnaire (*P *= 0.042) and between APA levels versus tiredness (*P *= 0.003).

Patients with positive APA levels showed a lower percentage of constipation (26% versus 42%, *P *< 0.05) and a higher percentage of sore throat (43.5% versus 24.4%, *P *< 0.05).

**Figure 2:**
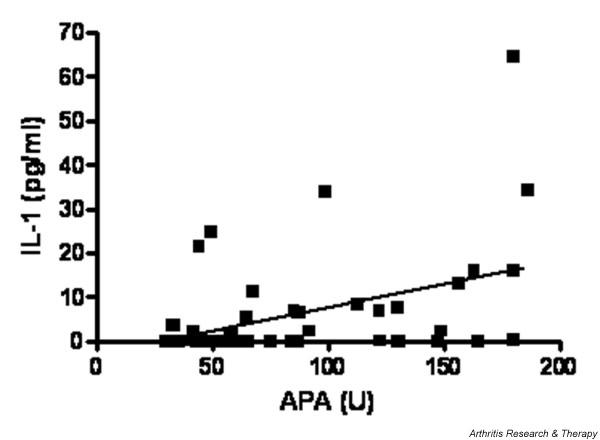
Correlation between antipolymer antibody and IL-1 levels. Correlation between antipolymer antibody (APA) and IL-1 levels in the subgroup of fibromyalgia patients with APA >30 U.

No differences in cytokine levels were detected between FM patients with mild, moderate or severe symptoms or between those FM patients with APA-positive/negative values. A significant correlation was found, however, between APA levels and IL-1 values within the subset of patients with positive APA (*P *= 0.0072; Figure 2).

APA seroreactivity results were low (7.7%) in the autoimmune noncase group.

## Discussion

The production of APA antibodies may result from an immunological response to self-antigens or to environmental agents, such as medications, silicone, or other chemicals, which results in the formation of antibodies cross-reacting with partially polymerized acrylamide. Because it has been shown that APA antibodies are present in about 50% of patients with silicone breast implants, and the highest prevalence of APA is found in implant patients with severe symptoms of a FM-like syndrome [8], subjects with a history of silicone gel-filled breast implants or breast surgery were excluded from the present study.

Our objective was to examine the presence of APA seropositivity in 285 nonimplanted FM patients and to verify whether APA levels correlate with disease severity and with cytokine levels.

We did not find differences of seropositivity between FM patients and control individuals but did observe that patients with moderate and severe symptoms exhibited qualitatively higher levels of APA, even if not statistically significant, with respect to patients with mild symptoms. Lee and colleagues [11] also did not find differences between Korean FM patients and control individuals, but reported a lower percentage of seropositivity (7.2%). Wilson and colleagues [9], on the contrary, studying an American population, found a higher percentage of seropositivity in FM patients (47%) while the percentage reported for control individuals was similar to ours.

Wilson and colleagues [9], like the present study, observed higher APA levels with the presence of more severe symptoms, while other authors [11] showed a downward trend as the symptom severity increased.

Interestingly, in our study the APA levels correlated with Fibromyalgia Impact Questionnaire estimates and tiredness results in the subset of seropositive patients, so APA correlated with the severity of the disease only when it is positive. This is the first study reporting an association between APA seropositivity and the Fibromyalgia Impact Questionnaire.

Like other reports [10,11], we found that the APA assay was negatively associated with age, probably because APA is not associated with autoimmune diseases and with a T-helper-2 response. We found a positive correlation between APA and IL-1 levels in the patient subgroup with APA >30 U, which might be associated with an immunological response to environmental agents in some way related to polymerized acrylamide. The higher percentage of sore throat in the seropositive patients might in some way be linked to this response. This possible association is further strengthened by previous observations of elevated levels of APA in patients with infection-associated malfunctions of silicone-based ventriculoperitoneal shunts [17].

We found a low prevalence of APA seroactivity in the noncase group that is about one-half of that found in FM patients. This result is in accordance with a precedent work [8], re-emphasizing the thesis that APA is not a general marker for autoimmune disease process.

## Conclusion

We have shown that APA cannot be considered a marker of disease in Italian FM patients. Its presence, however, might permit the identification of a subset of FM patients with more severe symptoms and who may respond differently to different therapeutic strategies. APA seroactivity is also not a general marker for autoimmune disease processes.

## Abbreviations

APA = antipolymer antibody; ELISA = enzyme-linked immunosorbent assay; FM = fibromyalgia; IL = interleukin; TNF = tumour necrosis factor.

## Competing interests

The authors declare that they have no competing interests.

## Authors' contributions

LB conceived of the study, its design and coordination, evaluated the clinical parameters and helped to draft the manuscript. CG, FDF and TG performed the laboratory assays. AR performed the statistical analysis and drafted the manuscript. MD and CT participated in the evaluation of clinical parameters. RBW provided the kit and helped to draft the manuscript. SB participated in the design of the study. All authors read and approved the final manuscript.
